# Impact of negative attitudes and information-seeking behavior of adults with type 2 diabetes on treatment in Japan

**DOI:** 10.3389/fpubh.2025.1645281

**Published:** 2025-09-18

**Authors:** Takako Mohri, Sawako Okamoto, Tomoaki Imamura, Hitoshi Ishii

**Affiliations:** 1 Department of Diabetes and Endocrinology, Nara Medical University, Kashihara, Nara, Japan; 2 Department of Public Health, Health Management and Policy, Nara Medical University, Kashihara, Nara, Japan; 3 Department of Doctor-Patient Relationships, Nara Medical University, Kashihara, Nara, Japan

**Keywords:** diabetes mellitus, adherence, information-seeking behavior, self-management, HbA1c

## Abstract

**Introduction:**

Effective self-management of type 2 diabetes mellitus in adults is influenced by emotions, thoughts, and information-seeking behavior. This study examined the relationship between the negative attitudes of Japanese adults with type 2 diabetes mellitus and information-seeking behavior, and whether this is associated with lower HbA1c levels.

**Methods:**

Adult outpatients with type 2 diabetes mellitus completed a questionnaire survey and the relationships between negative attitudes at diagnosis and consultation, information-seeking behavior, and HbA1c levels were examined.

**Results:**

Respondents with higher HbA1c tended not to seek information (*p* = 0.04) and felt they could not focus on work or study (*p* = 0.02). There was a significant association between respondents who agreed that “It's my fault that things go wrong” and “I took a long time before seeing a doctor after diabetes mellitus was suspected” (*p* = 0.04). Positive responses to “It's my fault that things go wrong,” “I worry about how I appear to others,” and “I feel it's undignified to ask others for help” were associated with information-seeking behavior (*p* = 0.03, *p* = 0.02, and *p* = 0.01, respectively). Analyses including interactions showed that patients who delayed seeing a doctor and agreed with “It's my fault that things go wrong” adopted information-seeking behavior (*p* = 0.02).

**Discussion:**

It appears that, even with negative attitudes, the combination of a long time before seeing a doctor and a self-blame mindset was associated with information-seeking behavior. This suggests that, even in type 2 diabetes mellitus patients who have negative attitudes, their self-management behavior can improve if their mindset leads to information-seeking behavior.

## Introduction

The number of people with diabetes mellitus (DM) has continued to increase globally and the World Health Organization (WHO) considers it a major public health challenge. According to estimates by the International Diabetes Federation (IDF), the age-standardized diabetes prevalence rate for people aged 20–79 worldwide in 2024 was 11.1%, with higher rates in urban areas (12.7%). In the western Pacific region, which includes Japan, the diabetes prevalence rate is the third highest in the world (12.4%), with Japan at 8.1%. In 2024, Japan's population with diabetes was the 10th highest globally (10.8 million), declining from its 9th place position in 2021 (1). DM is treatable and factors including medication, regular screening and treatment for complications can mitigate or delay its consequences. It is also important for the patient to follow a healthy diet, exercise regularly, maintain a normal body weight and avoid tobacco use (2). Thus, proactive involvement in the treatment of individuals with DM is essential.

In particular, type 2 DM (T2DM) is the most common form of diabetes, accounting for over 96% of all diabetes cases worldwide, and is prevalent among the older adults (1–3). The age-standardized prevalence of T2DM worldwide in 2021 was 5.9%, with Japan at approximately 5.6%, a relatively high rate (3). By contrast, in 2017 the age-standardized incidence rate was relatively low, at 177 per 100,000 in Japan, compared to 279 per 100,000 worldwide (4).

T2DM is defined as a metabolic disorder characterized by relative insulin deficiency due to impaired insulin secretion and insulin sensitivity, leading to chronic hyperglycemia (5). It is diagnosed by excluding type 1 diabetes and other types (diabetes with identified genetic abnormalities, diabetes associated with other diseases or conditions) (5, 6). Although often asymptomatic, it increases the risk of myocardial infarction by 84%, stroke by 54%, and vascular dementia by 103% in addition to microvascular disease (1). T2DM can be prevented or its progression delayed through lifestyle improvements (1). In Japan, HbA1c measurement is commonly used as an indicator of blood glucose management to confirm the effectiveness of lifestyle improvements and treatment. It is performed as part of a blood test during outpatient visits, and monthly HbA1c measurement is covered by health insurance. Although HbA1c has its limitations as an indicator of treatment efficacy and disease status, several studies have used it as the indicator, and clinically meaningful results have been reported (7–10). In Japan, the target value for preventing complications is set at < 7.0% HbA1c (11). For older adults people aged 75 years or older without dementia and those taking medications that pose a risk of severe hypoglycemia, maintaining HbA1c levels below 8% is recommended (12).

T2DM patients are required to engage in continuous self-management, such as diet and exercise, because prolonged hyperglycemia increases the risk of various complications. This is considered to be a major psychological burden, with approximately one-third of patients reporting mental distress (13). This burden seems to be one factor contributing to the high incidence of anxiety disorders and depression (14). In addition, the stigma associated with being diagnosed with diabetes, which is similar to the discrimination imposed by society, also contributes to the mental distress of patients. In Japan, due to this stigma, 50.6% of people with T2DM do not disclose their condition to their superiors at work, and some do not receive appropriate treatment (15).

Poor adherence of adults with T2DM affects treatment (16) and can lead to increased stress for doctors if there are unfavorable outcomes (17). Therefore, positive health behaviors have been promoted using various strategies on the medical provider's side such as diabetes self-management education, nutrition therapy, physical activity, and psychosocial care (7). Of these, improving self-management appears to be associated with lower HbA1c levels (16, 18–21). As such, the American Diabetes Association (ADA) strongly recommends that people with DM participate in diabetes self-management education and support (DSMES) (7). However, how to motivate people with T2DM to become proactively involved in their self-management has yet to be elucidated.

Whether and how adults with T2DM acquire knowledge about their condition are important factors in self-management behavior (21, 22). As a part of this behavior, medication adherence is closely related to the patient's motivation to search for information to acquire knowledge. More active information-seeking behavior is associated with better medication adherence (22–25). This includes obtaining information from medical professionals as well as independently searching for, and incorporating, information from other sources (22, 24–26). There are a limited number of academic reports regarding information-seeking behavior, and it is also necessary to examine whether information-seeking behavior would be a cue to motivate self-management behavior in Japan.

The ADA recommends that the monitoring of self-management behaviors and their impact on clinical outcomes, health status, and quality of life (QOL), as well as the psychosocial factors that affect a patient's ability to self-manage, be part of routine clinical care. Furthermore, health-related QOL and health outcomes should be optimized by providing psychosocial care for problems arising from diabetes such as diabetes distress, anxiety, and poor sleep hygiene (7).

From the perspective of overall healthcare, among the inherent properties of patients, the D-type personality is known to be a risk factor for widespread adverse health effects. The concept of the D-type personality has two characteristics, negative affectivity (NA) and social inhibition (SI). NA refers to the tendency to be unable to take a positive attitude toward oneself and often evokes negative responses such as anxiety and depression (27). Those displaying SI tend to suppress emotional expression in social situations and not seek support from others to avoid antipathy (28). In people with DM, the D-type personality has been reported to be associated with unhealthy behavior that is not directly associated with HbA1c levels but is unresponsive to treatment (29). Conversely, other reports have stated that the D-type personality directly affects both QOL and metabolic control (HbA1c) (30, 31).

In Japan, universal health insurance is in place, and medical care, including consultations with specialists, is relatively accessible. However, delays in seeking medical care still occur. Local governments their healthcare related facilities and have provided telephone follow-up programs that remind people to see a DM specialist and explain medical conditions for those who did not see their medical doctors. However, the tendency to delay seeing their doctors or a DM specialist has not been improved. Initially, it seemed that adults with T2DM were not mentally prepared to see their doctors, but subsequent reports have shown that this is not the case, and the reason for this remains unclear in Japan (32).

Crucially, the reactions that adults with T2DM have at the time of diagnosis are difficult to change even after the passage of time (33) and it remains unclear how the distress at the time of diagnosis and subsequent consultations influences the self-management behavior of people with T2DM.

The purpose of this study was to clarify whether the information- and support-seeking behaviors, negative attitudes, reactions at the time of diagnosis and at subsequent consultations of Japanese adults with T2DM are related to each other and to lower HbA1c levels.

## Materials and methods

### Research design and sample selection

This was a cross-sectional study. A questionnaire survey was conducted from July 1, 2015 to February 19, 2016 at the DM Center of the university-affiliated hospital where the authors work. The participants were 121 outpatients with DM aged 20 years or older who were treated by one of the authors.

### Inclusion and exclusion criteria

Patients were included if they had no visual impairment, paralysis, or severe dementia and were able to complete the questionnaire themselves. Adults with gestational DM, type 1DM, DM caused by other diseases, pregnant women with T2DM, and patients for whom HbA1c values were not useful as an indicator due to concomitant diseases were excluded.

### Questionnaire design

The questionnaire was created with reference to previous studies (1–33). Question items regarding information-seeking motivation and behaviors and understanding of complications were created since information seeking is closely related to self-management (20, 22–24).

Question items regarding reactions at the time of diagnosis and attitudes at the consultations were created. Negative attitudes of adults with T2DM have an effect on treatment and self-management so question items regarding inherent negative attitudes, diabetes distress, anxiety, and sleep health were included (33–36).

Furthermore, adults with T2DM who are concerned about how they appear to others tend to worry greatly and avoid social interaction because of a fear of embarrassing themselves. These attitudes can potentially lead to the patient not asking for help, which influences their treatment and attitudes toward treatment (37). To address this, question items for “Patient's support-seeking attitudes,” such as being able to ask for help and to listen to them, feeling it is undignified to ask others for help, and being concerned about how they appear to others were created (Supplementary Table S1). Additionally, a question about the desire to discuss matters unrelated to DM at consultations was included in this group. Those who chat freely at a consultation but not so much about their treatment might be reluctant to accept their DM and/or its treatment or try to avoid any task which a patient should be able to tackle without asking for help from others (38).

### Data collection

After a routine consultation, the doctor who is one of the authors raised the matter of the questionnaire. If the patients showed an interest and indicated willingness to participate, after obtaining their permission to explain our study, the doctor provided an explanation and asked them to participate. Their right to withdraw at any time, even after completing the survey, was explained and they were provided with a consent withdrawal form along with an explanatory document to ensure they were able to withdraw their consent later if necessary.

They were also informed that only this doctor was allowed to access their medical records. They were asked to anonymously provide, from their medical charts, their T2DM history, HbA1c level at the time of the survey, complications, and other illnesses if they consented to participate. Other researchers/authors of this study were able to access only the anonymized data. The authors in this study analyzed the results of the questionnaire survey together with these clinical data.

In this study, HbA1c was used as an objective indicator. HbA1c is considered to be a longitudinal parameter and a reliable test (39–41), can be measured during patient visits, and has been used in other studies (7–10). The target HbA1c level is generally < 7% to prevent the development of microvascular complications (39). However, it is individualized and affected by factors such as age, cognitive function, the use of drugs that may cause severe hypoglycemia, activities of daily living, and comorbidities. Although there are limitations to this approach (39), HbA1c is associated with various pathological conditions and certain limited treatment results.

### Statistical tests

Factor analysis and reliability testing (Cronbach's α) were initially conducted to confirm the validity of the questionnaire survey data.

Attributive variables included sex, age, household income, disease duration, the period from the time the participant was first diagnosed or suspected of having T2DM at a health checkup until they consulted a doctor about it, and HbA1c values, which were used as independent variables. Logistic regression analysis was performed for each question item as the dependent variable.

In the Japanese health insurance system, there are two types of insurance: one for those aged 75 years and over and another for those under 75 years. Furthermore, one particular health checkup focuses on metabolic syndrome, which raises the risk of diabetes, and has been required for all those aged 40–74 since 2008 in Japan. Therefore, the participants were divided into the following age groups: 75 years and over, 74–65, 64–55, 54–45, and 44 years and under.

The HbA1c measurements were divided into five sub-groups: 6.9 and under, 7.0–7.9, 8.0–8.9, 9.0–9.9, and 10 and over. Additionally, regarding information-seeking behavior, we conducted analyses stratified by HbA1c levels at 8% to make sure whether the results are different depending on the HbA1c value.

Regarding the scale of responses to questions, a 6-point, Likert scale was used with 1 being “not at all” and 6 being “very much.” In psychological statistics, variables numbering six or more for a question can be analyzed as numeric variables (42). For subsequent analyses, the responses were converted into dichotomous variables with 1 to 3 as 0 and 4 to 6 as 1. By using this method, it is possible to overcome the Japanese tendency to select the median value in surveys (43). Correlation and regression analysis, together with multiple logistic regression analyses were performed.

To examine the relationships among the dependent variables, each of the dependent variables that had a significant association with the independent variables in the previous step of the analysis was added as an additional independent variable. All analyses were performed using SPSS version 25.

### Ethical permission

This study was approved by the Nara Medical University Ethics Review Committee (#1003).

## Results

Of the 121 questionnaires distributed, 119 (98.3%) responses were obtained, of which 98 (81.0%) were valid. Of the 21 adults with DM who were excluded, 19 had diseases other than T2DM (5 gestational DM, 10 type 1 DM, and 4 with other diseases), one was pregnant, and one patient had an HbA1c value that was not useful as an indicator due to comorbidity. The mean age of the participants (*n* = 98) was 63.6 ± 11.3 (range 32–83) years, and there were 70 men (71.4%) and 28 women (28.6%). The mean HbA1c (National Glycohemoglobin Standardization Program: NGSP) value was 7.9 ± 1.3% (5.6%−12.1%), and the mean duration of T2DM disease was 12.8 ± 8.5 years. Regarding the time it took to see a doctor for T2DM, about half of the respondents visited a medical institution within 6 months, and 10% took more than 5 years (Table 1).

**Table 1:** Demographics of participants.

**Demographic items**	***n* = 98**
Male, *n* (%)	70 (71.4)
Age (years)	63.6 ± 11.3
HbA1c (NGSP %)	7.9 ± 1.3
Duration of diabetes (years)	12.8 ± 8.5
Microvascular complication: (presence %)	62 (63.3)
Neuropathy	52 (53.1)
Nephropathy	43 (43.9)
Retinopathy	32 (32.7)
Comorbidities (presence %)	83 (84.7)
**Period until outpatient consultation (months; %;** ***n*** = **82)**	
To 6 months	48 (49.0)
From 6 months to 1 year	3 (3.1)
From 1 to 3 years	13 (13.3)
From 3 to 5 years	8 (8.2)
From 5 years	10 (10.2)
Missing value	16 (16.3)
**Household income (yen** = ¥**; %;** ***n*** = **86)**	
Under ¥3 million	40 (40.8)
¥3 million-under 4 million	13 (13.3)
¥4 million-under 5.5 million	12 (12.2)
¥5.5 million-under 7 million	8 (8.2)
¥7 million or more	13 (13.3)
Missing value	12 (12.2)

Data are presented as number (%) or mean ± SD. $1 = ¥157.7, May 31, 2024.

Among those with T2DM microvascular complications (*n* = 62), 53.1% had neuropathy, of which 3% (*n* = 3) were at stage 4 or higher. In addition, 84.7% (*n* = 83) had comorbidity, where cancer 18.3% (*n* = 18), and macrovascular complications 7.1% (*n* = 7), and other diseases requiring steroid use 14.3% (*n* = 14) were the top three conditions.

As a result of factor analysis, five factors were identified and used as dependent variables: tendency of patient's information seeking, patient's reactions at a doctor's consultation, patient's negative attitudes, tendency of seeking support, and patient's reactions at the time of diagnosis. The validity and internal consistency of the factor analysis was confirmed (Cronbach's α = 0.72; Table 2 and Supplementary Table S2).

**Table 2:** Questionnaire and factor analysis.

	**Questionnaire**	**Factor analysis**				
Patient's information seeking motivation/action	Group I) tendency of patient's information seeking					
	Want to know about diabetic complications; motivation	0.96^*^				
	Want to read documentation about complications; motivation	0.83^*^				
	Possibility of developing complications; comprehension	0.52^*^				
	Information-seeking behavior	0.43^*^			0.39	
Patient's reactions at consultation	Group II) patient's reactions at a doctor's consultation					
	Scared		0.9^*^		0.39	
	Feel down		0.58^*^			
	Feel better that doctor listens		−0.46^*^			
Patient's inherent negative attitudes	Group III) tendency of patients to dwell on the inherent negatives					
	(Patient's negative attitudes)					
	Things going wrong are my fault			0.63^*^		
	Can focus on work or study			−0.61^*^		
	Anxiety about diabetes			0.55^*^		
	Can sleep well		−0.36	−0.42^*^		
Patient's support-seeking attitudes	Group IV) tendency of patients to seek support from others					
	(Tendency of seeking support)					
	Undignified to ask others for help				0.64^*^	
	Desire to discuss matters unrelated to DM				0.56^*^	
	Asking people other than my regular health providers to listen to my stories				0.54^*^	
	Concerned about how I appear to others				0.31^*^	
Patient's reactions at diagnosis	Group V) patient's reactions at the time of diagnosis					
	Do my best to comply with the treatment					0.60^*^
	I knew it					0.57^*^
	Uneasy about the future		0.39			0.43^*^

Cronbach's α = 0.72. The word “patient” in this table means “adult with T2DM.” ^*^Values of factor loadings in the attributed domain. Abbreviations of each group are indicated in parentheses. The abbreviations are used in other tables and a figure in this study.

### The relationship between attributive variables, information- and support-seeking and inherent negative attitudes

**Figure 1:**
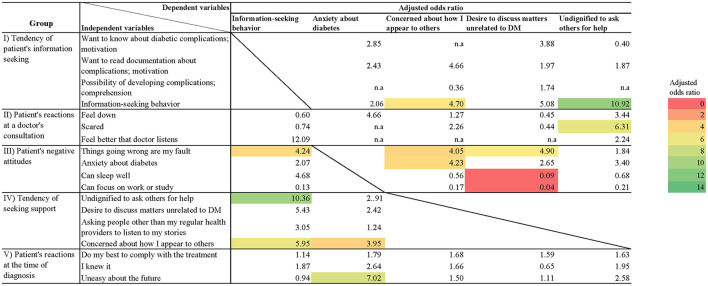
Heatmap of adjusted odds ratios for each question item. Shows adjusted odds ratios for questions with significant differences. Questions with no significant differences are not colored.

Respondents with high HbA1c levels tended not to seek information (*p* = 0.04) and felt that they were unable to focus on work or study (*p* = 0.02; Supplementary Table S3, Figure 1, Supplementary Figures S1, S2). Those who answered, “I feel it's undignified to ask others for help,” “It's my fault that things go wrong” and “I'm concerned how I appear to others” adopted information-seeking behavior (*p* = 0.01, *p* = 0.03, *p* = 0.02, respectively; Table 3, Supplementary Table S4; Supplementary Figure S3). Additionally, in the analysis stratified by HbA1c levels at 8%, those who stated they “Feel better that a doctor listens” and had a “Desire to discuss matters unrelated to DM” also engaged in information-seeking behavior (*p* = 0.04, adjusted odds ratio 14.29, 95% CI (1.13–180.35); *p* = 0.04, adjusted odds ratio 5.64, 95% CI (1.08–29.53), respectively; Supplementary Table S5). Compared with the results of analysis that did not stratify at 8% (Supplementary Table S4), the direction of the odds ratios of both analyses were consistent. The only question for which the direction of the odds ratios was inconsistent was “I felt anxious about the future at the time of diagnosis” but no significant difference was found. Although the possibility of differences in the effects depending on metabolic status was suggested, the sample size was insufficient, and further studies are needed. Respondents who took a long time to see a doctor for T2DM significantly believed that it was their fault that things went wrong (*p* = 0.04; Supplementary Table S3), and those who engaged in information-seeking behavior were associated with lower HbA1c values (*p* = 0.04; Supplementary Table S3).

**Table 3:** Relationship between patient's information-seeking behavior and patient's attitudes.

**Group**	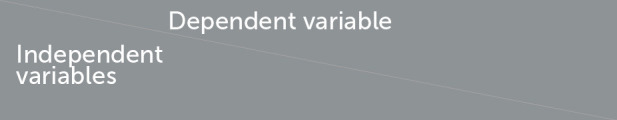	**Information-seeking behavior**	
		***p*** **-Value**	**Adjusted odds ratio (95% CI)**
II) Patient's reactions at a doctor's consultation	Feel down	0.51	0.60 (0.13–2.72)
	Scared	0.72	0.74 (0.15–3.71)
	Feel better that doctor listens	0.06	12.09 (0.95–154.12)
III) Patient's negative attitudes	Things going wrong are my fault	0.03	4.24 (1.13–15.87)^*^
	Anxiety about diabetes	0.27	2.07 (0.58–7.46)
	Can sleep well	0.12	4.68 (0.65–33.47)
	Can focus on work or study	0.11	0.13 (0.01–1.60)
IV) Tendency of seeking support	Undignified to ask others for help	0.01	10.36 (1.95–54.99)^*^
	Desire to discuss matters unrelated to DM	0.05	5.43 (1.01–29.32)
	Asking people other than my regular health providers to listen to my stories	0.14	3.05 (0.69–13.51)
	Concerned about how I appear to others	0.02	5.95 (1.35–26.23)^*^
V) Patient's reactions at the time of diagnosis	Do my best to comply with the treatment	0.86	1.14 (0.27–4.80)
	I knew it	0.37	1.87 (0.48–7.26)
	Uneasy about the future	0.92	0.94 (0.27–3.23)

^*^p < 0.05. The dependent variable in each case in this table is information-seeking behavior. The independent variables as adjustment factors, sex, age, household income, period until outpatient consultation, duration of diabetes, and HbA1c, were analyzed at each step together with the specified additional independent variable as shown in each line of the table. The results are listed without adjustment factors. See Supplementary Table S4 for details.

Those who were concerned about how they appear to others were significantly associated with the statements, “It's my fault that things go wrong” and “I feel anxious when I think about my diabetes” (*p* = 0.01, *p* = 0.02, respectively; Table 4, Supplementary Table S6).

**Table 4:** Relationship between “Concerned about how I appear to others” and patient's other attitudes.

**Group**	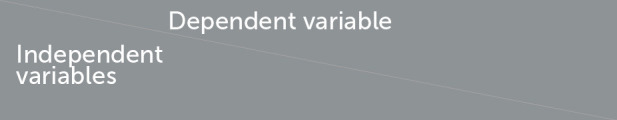	**Concerned about how I appear to others**	
		***p*** **-Value**	**Adjusted odds ratio (95% CI)**
I) Tendency of patient's information seeking	Want to know about diabetic complications; motivation	n.a	
	Want to read documentation about complications; motivation	0.09	4.66 (0.77–28.25)
	Possibility of developing complications; comprehension	0.43	0.36 (0.03–4.65)
	Information-seeking behavior	0.03	4.70 (1.21–18.20)^*^
II) Patient's reactions at a doctor's consultation	Feel down	0.76	1.27 (0.29–5.61)
	Scared	0.35	2.26 (0.41–12.46)
	Feel better that doctor listens	n.a	
III) Patient's negative attitudes	Things going wrong are my fault	0.01	4.05 (1.35–12.12)^*^
	Anxiety about diabetes	0.02	4.23 (1.22–14.7)^*^
	Can sleep well	0.53	0.56 (0.09–3.52)
	Can focus on work or study	0.07	0.17 (0.03–1.19)
V) Patient's reactions at the time of diagnosis	Do my best to comply with the treatment	0.45	1.68 (0.43–6.55)
	I knew it	0.39	1.66 (0.52–5.35)
	Uneasy about the future	0.46	1.50 (0.52–4.35)

^*^p < 0.05, n.a: no answer. The dependent variable in each case in this table is “Concerned about how I appear to others.” The independent variables, sex, age, household income, period until outpatient consultation, duration of diabetes, and HbA1c, were analyzed at each step together with the specified additional independent variable as shown in each line of the table. The results are listed without adjustment factors. See Supplementary Table S6 for details.

Those who wanted their doctor to listen to them about matters unrelated to T2DM and its treatment were significantly associated with the statements, “It's my fault that things go wrong,” “If I think about DM, I won't be able to focus on work or study” and “If I think about DM, I won't be able to sleep” (*p* = 0.02, *p* < 0.01, *p* = 0.02, respectively; Table 5).

**Table 5:** Relationship between patient's desire to discuss “matters unrelated to DM” and other attitudes.

**Group**	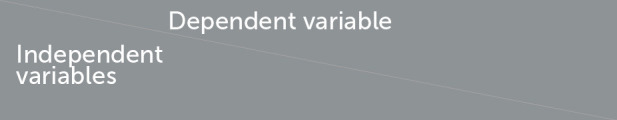	**Desire to discuss matters unrelated to DM**	
		***p*** **-Value**	**Adjusted odds ratio (95% CI)**
I) Tendency of patient's information seeking	Want to know about diabetic complications; motivation Want to read documentation about complications; motivation Possibility of developing complications; comprehension	0.24	3.88 (0.41–36.46)
		0.44	1.97 (0.35–11.04)
		0.66	1.74 (0.15–20.26)
	Information-seeking behavior	0.05	5.08 (1.00–25.89)
II) Patient's reactions at a doctor's consultation	Feel down	0.35	0.45 (0.08–2.40)
	Scared	0.34	0.44 (0.08–2.40)
	Feel better that doctor listens	n.a	
III) Patient's negative attitudes	Things going wrong are my fault	0.02	4.90 (1.36–17.69)^*^
	Anxiety about diabetes	0.16	2.65 (0.69–10.23)
	Can sleep well	0.02	0.09 (0.01–0.65)^*^
	Can focus on work or study	< 0.01	0.04 (0.01–0.33)^*^
V) Patient's reactions at the time of diagnosis	Do my best to comply with the treatment	0.54	1.59 (0.36–6.97)
	I knew it	0.46	0.65 (0.21–2.05)
	Uneasy about the future	0.86	1.11 (0.38–3.24)

^*^p < 0.05, n.a: no answer. The dependent variable in each case in this table is “Desire to discuss matters unrelated to DM.” The independent variables, sex, age, household income, period until outpatient consultation, duration of diabetes, and HbA1c, were analyzed at each step together with the specified additional independent variable as shown in each line of the table. The results are listed without adjustment factors. See Supplementary Table S7 for details.

In the analysis including interactions, those who waited a long time before consultation and respondents with high HbA1c values were significantly not engaging in information-seeking behavior (*p* = 0.03, *p* = 0.03, respectively; Table 6). However, respondents who thought it was their fault that things went wrong and took a long time to see a doctor were significantly more likely to seek information (*p* = 0.02; Table 6, Supplementary Figure S3). Respondents who felt that it was undignified to ask others for help and had anxiety about diabetes, as well as those who felt concerned about how they appeared to others and had anxiety about diabetes showed a significantly stronger tendency to engage in information-seeking behavior (*p* = 0.02, *p* = 0.02, respectively; Supplementary Tables S8, S9).

**Table 6:** Examination of interactions between information-seeking behavior and attributive variables including an interactive item.

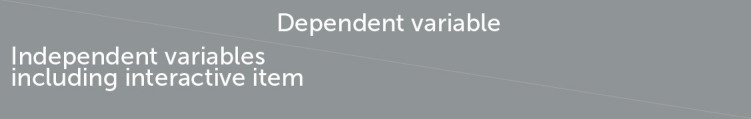	**Information-seeking behavior**	
	***p*** **-Value**	**Adjusted odds ratio (95% CI)**
Sex	0.19	0.28 (0.04–1.86)
Age	0.35	1.40 (0.70–2.80)
Household income	0.65	1.11 (0.70–1.76)
Period until outpatient consultation (months)	0.03	0.38 (0.16–0.93)^*^
Duration of diabetes (years)	0.28	0.78 (0.50–1.22)
HbA1c	0.03	0.48 (0.25–0.92)^*^
Things going wrong are my fault	0.30	0.27 (0.02–3.23)
Things going wrong are my fault × Period until outpatient consultation (months)	0.02	3.87 (1.24–12.08)^*^

^*^p < 0.05.

### Relationship between patient reactions at the time of diagnosis and consultation and patient's attitudes

Respondents who were uneasy about the future at the time of diagnosis were significantly more likely to answer, “I feel anxious when I think about my diabetes” (*p* < 0.01; Table 7). Respondents who felt it was undignified to ask others for help were significantly more likely to answer “I'm scared to talk to my doctor” at the time of consultation (*p* = 0.04; Table 8). There were no statistically significant results other than these regarding reactions at the time of diagnosis and consultation for the purposes of this study.

**Table 7:** Relationship between patient's anxiety about diabetes and other attitudes.

**Group**	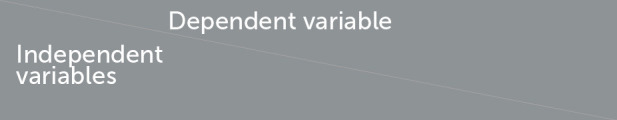	**Anxiety about diabetes**	
		***p*** **-Value**	**Adjusted odds ratio (95% CI)**
I) Tendency of patient's information seeking	Want to know about diabetic complications; motivation	0.25	2.85 (0.49–16.64)
	Want to read documentation about complications; motivation	0.27	2.43 (0.50–11.72)
	Possibility of developing complications; comprehension	n.a	
	Information-seeking behavior	0.25	2.06 (0.60–7.16)
II) Patient's reactions at a doctor's consultation	Feel down	0.18	4.66 (0.50–43.57)
	Scared	n.a	
	Feel better that doctor listens	n.a	
IV) Tendency of seeking support	Asking people other than my regular health providers to listen to my stories	0.74	1.24 (0.35–4.39)
	Undignified to ask others for help	0.08	2.91 (0.88–9.57)
	Concerned about how I appear to others	0.03	3.95 (1.16–13.42)^*^
	Desire to discuss matters unrelated to DM	0.19	2.42 (0.65–9.01)
V) Patient's reactions at the time of diagnosis	Do my best to comply with the treatment	0.41	1.79 (0.45–7.05)
	I knew it	0.13	2.64 (0.75–9.26)
	Uneasy about the future	< 0.01	7.02 (1.93–25.56)^*^

^*^p < 0.05, n.a: no answer. The dependent variable in each case in this table is “Anxiety about diabetes.” The independent variables, sex, age, household income, period until outpatient consultation, duration of diabetes, and HbA1c, were analyzed at each step together with the specified additional independent variable as shown in each line of the table. The results are listed without adjustment factors. See Supplementary Table S10 for details.

**Table 8:** Relationship between patient's believing that it is “undignified to ask others for help” and other attitudes.

**Group**	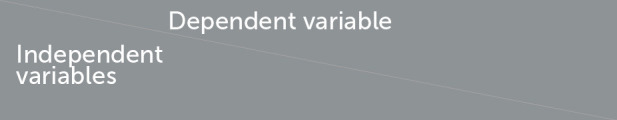	**Undignified to ask others for help**	
		***p*** **-Value**	**Adjusted odds ratio (95% CI)**
I) Tendency of patient's information seeking	Want to know about diabetic complications; motivation	0.33	0.40 (0.06–2.55)
	Want to read documentation about complications; motivation	0.45	1.87 (0.37–9.41)
	Possibility of developing complications; comprehension	n.a	
	Information-seeking behavior	0.01	10.92 (2.05–58.19)^*^
II) Patient's reactions at a doctor's consultation	Feel down	0.11	3.44 (0.77–15.38)
	Scared	0.04	6.31 (1.05–37.94)^*^
	Feel better that doctor listens	0.52	2.24 (0.20–25.77)
III) Patient's negative attitudes	Things going wrong are my fault	0.26	1.84 (0.64–5.36)
	Anxiety about diabetes	0.06	3.40 (0.97–11.85)
	Can sleep well	0.65	0.68 (0.13–3.65)
	Can focus on work or study	0.08	0.21 (0.03–1.24)
V) Patient's reactions at the time of diagnosis	Do my best to comply with the treatment	0.48	1.63 (0.42–6.31)
	I knew it	0.27	1.95 (0.60–6.31)
	Uneasy about the future	0.09	2.58 (0.86–7.73)

^*^p < 0.05, n.a: no answer. The dependent variable in each case in this table is “Undignified to ask others for help.” The independent variables, sex, age, household income, period until outpatient consultation, duration of diabetes, and HbA1c, were analyzed at each step together with the specified additional independent variable as shown in each line of the table. The results are listed without adjustment factors. See Supplementary Table S11 for details.

## Discussion

The purpose of this study was to clarify how negative attitudes of adults with T2DM in Japan and their reactions at the times of diagnosis and consultation are related to their willingness to seek information and their behavior, and whether they are associated with lower HbA1c levels.

There was an association between high HbA1c levels and a tendency not to engage in information-seeking behavior and an inability to focus on work or study when thinking about T2DM. On the other hand, it was also revealed that even if Japanese adults with T2DM had negative attitudes, those who were able to engage in information-seeking behavior did not have elevated HbA1c levels.

### The relationship between negative attitudes and information- and support-seeking behaviors of Japanese adults with T2DM

Adults with T2DM who have negative attitudes toward their condition are more likely to engage in unhealthy behaviors and not seek information (25, 26, 36). The results of this study also showed that negative attitudes which are inherent and/or acquired after T2DM diagnosis, such as blaming oneself when things go wrong and feeling anxious when thinking about T2DM, tended to be associated with non-support-seeking attitudes, such as feeling it's undignified to ask others for help, concern for how they appear to others, and a desire for doctors to discuss matters unrelated to T2DM and its treatment at a consultation.

However, we found in this study that even if Japanese adults with T2DM have negative and/or non-support-seeking attitudes, some of them also tended to engage in information-seeking behavior. Participants in this study who took a long time to see a doctor for their condition did not engage in information-seeking behavior. A negative attitude like believing it was their fault that things go wrong can be said to be a self-blame thought (44, 45). Some who had these thoughts and took a long time to see a doctor sought information.

Respondents seemed to fill the perceived gaps in their knowledge by seeking information and solving the problem on their own instead of asking others for help. This is in accordance with the findings of previous studies (21, 44–46). The concept of intropersistiveness in the Picture-Frustration (PF) Study scoring elements tends to drive those who blame themselves to solve their own problems (44–46). The PF study is a psychological test that analyzes responses to frustrating situations and assesses an individual's personality traits and interpersonal adaptability. It has also been shown that a patient's psychological characteristics regarding frustrating situations are reflected in their diabetes treatment behavior (45). The analysis includes three types of aggression responses: “other-blaming, self-blame, and no-blame” and classification into disorder-significant, ego-defensive, and demand-persistent responses (44). Combining these factors, the results of this study may indicate a reaction similar to an intropersistive response. An intropersistive response is a response that attempts to solve problems in order to relieve stress. The results of the present study suggest that the participants may exhibit reactions similar to intropersistive reactions.

Self-critical tendencies arise not only from the importance placed on relationships and consideration for others in Japan, or from concerns about being evaluated by others, but also from the interdependence between viewing oneself critically and viewing others positively. It has been found that this includes mechanisms for involvement in groups and self-improvement, and the results suggest that cultural influences may be involved (46). Our results suggest that T2DM patients with self-blame thoughts and information-seeking behavior were motivated to increase their knowledge, and this might be associated with better self-management and lower HbA1c values, despite them taking longer to consult a doctor for T2DM. In East Asian cultures, including Japan, a mutually cooperative view of self is dominant (46). These findings may be generalizable to other East Asian communities with similar cultural characteristics. We also believe that this will be useful as a reference for countries with residents or immigrants who have cultures from these regions in the world.

It is clinically necessary for patients suspected of having T2DM to see a doctor as soon as possible after a referral. Medical professionals need to accept these patients' proactive attitudes toward solving the problem on their own, and at the same time, consider prompting them to see a doctor as soon as possible. The potential involvement of social support networks, such as family, friends, and patient groups, may be useful when it comes to diabetic patients' consultation behavior. Because this study focused on patients' own thoughts and intentions, these factors were not investigated, but we plan to include social support network factors in future research.

### Relationship between patient reactions at the time of diagnosis and consultation and information-seeking behavior

The DAWN2 study recommended that the psychosocial difficulties of living with T2DM be screened at, and addressed immediately after, a diagnosis (32). In this study, participants who felt uneasy about the future at the time of diagnosis also tended to feel anxious when they thought about their diabetes in their ensuing lives.

Those who were scared to talk to a doctor at the time of consultation were associated with the negative attitude of feeling it's undignified to ask others for help and this appeared to relate to information-seeking behavior in this study. The difficulty of requesting help may be influenced by the fact that Japanese people tend to have low levels of trait-empathic concern for those in need. The results of this study may also be influenced by such social norms. In Japan, there is a tendency to interpret suffering as punishment for deviating from social norms and order (47). Some of the patients who have a sense of self-blame tend to have a proactive attitude toward information-seeking and engage in information-seeking behavior. They have difficulty expressing themselves or may feel uncomfortable with their doctor so they might try to solve their problems on their own (44–46). Although further research is required, information-seeking behavior would appear to help adults with T2DM to become informed patients, possibly helping them to effectively self-manage their care in Japan.

Some consideration should be given to how healthcare providers respond to patients with T2DM who exhibit negative attitudes. It might appear that the patient is deviating from the discussion during a consultation when such behavior is observed but it is necessary to reexamine how to respond in these situations. If a doctor feels it is ethically acceptable to wait a little longer and devote more time to them, it might improve the patient's self-management behavior by encouraging them to seek information. It may be useful to try providing knowledge about alternative foods that patients may be interested in when they want to eat something sweet, or to share evidence that aerobic exercise improves the prognosis of T2DM in patients with mental illness (48, 49).

Furthermore, among patients with poor metabolic status (HbA1c levels of 8% or higher), those who wanted to talk about topics unrelated to diabetes or had their feelings listened to during the examination were suggested to be more likely to engage in information-seeking behavior. The odds ratio direction is also consistent, and it may also be possible to identify patients whose physicians should change their approach to interacting with the patient. The only item for which the direction of the odds ratios was inconsistent was “I felt uneasy about the future at the time of diagnosis,” but no significant difference was found. Although the possibility of differences in the effects depending on metabolic status was suggested, the sample size was insufficient, and further studies are needed.

The results of this study showed that even adults with T2DM who have negative attitudes and reactions may engage in information-seeking behavior when they have a self-blame mindset and try to solve a problem on their own. This suggests that searching for information possibly helps adults with T2DM better understand their disease and the situation they are placed in, which might lead to better self-management behavior. This study, which focuses on improving the psychosocial aspects of living with diabetes, is consistent with the intent of previous research that emphasizes the importance of paying greater attention to the situational context in which the self-regulatory processes underlying self-management occur, and presents the usefulness of information-seeking behavior as an example of the situational context (21).

### Limitations

Collection of the questionnaires in this study was conducted by one doctor at one institution. While this might introduce some bias it has the advantage of eliminating inter-doctor differences in patient care. Future studies should adopt a multi-center approach to reduce this bias. Furthermore, there were only 121 patients recruited for 18 variables. Although 10 participants per variable is one factor in determining sample size (50), our total of 121 was possibly the largest that one doctor—to ensure consistency of the data—was able to achieve. In particular, the questions asked of patients in this study included items that are difficult to answer honestly without a strong doctor-patient relationship, such as patients' own negative thoughts, tendency to seek support, and reactions including emotions at the time of diagnosis and consultation. However, to our knowledge, there are no previous studies that have conducted this deep a questionnaire survey of 121 people. Our findings provided enough useful insights to examine any tendencies of T2DM patients. Regarding the scale of responses to the questions in this study, since there is a possibility that some information may be lost due to binomialization, further studies with a larger number of cases are necessary to examine the capacity response.

Furthermore, this study obtained data from self-reported questionnaires without objective behavioral indicators, so there is a possibility of reporting bias. In future studies, we would like to incorporate the collection of data such as online search logs to enhance the objectivity of such indicators.

We used HbA1c values in this study as an objective measure. Despite its limitations it can be assessed during patient visits, after their consultation, and is reliable (39). Several studies have used HbA1c (7–10) and reported certain clinically meaningful results.

Information-seeking behavior seems to be influenced by participants' education level, and therefore by their health literacy. Education levels, which may influence their self-management, were not investigated though annual income data were used as a substitute (51, 52). No significant differences were observed in information-seeking motivation, behavior, or understanding.

Similarly, no significant relationship between sex and support-seeking tendency was found but the participants were mainly men. From the perspective of who to ask for help, gender differences were observed even in requests for assistance from experts, but the difference was small (53). Future studies should change the questions to specify the target population. As support-seeking behavior may differ due to education level and between sexes, further studies that address these issues are required. In particular, the lack of female representation is a matter that should be addressed in future studies.

Patients with longer medical histories might have inaccurately reported the time elapsed before seeing a doctor, leading to a recall bias. In Japan, this depends on a patient's self-report since a referral is not always required to see a doctor. Future studies should attempt to identify patients with referrals so that diagnosis dates can be confirmed.

It is difficult to distinguish whether insomnia is a cause or effect in adults with T2DM. However, our questionnaire item asked about sleep as an anxiety question and not as a sleep disorder question, and because the data were extracted from medical records, we think it is acceptable to handle the responses as an anxiety item. The differences between questions about diseases and those about anxiety should be addressed in future studies.

Finally, in this study, the key question was whether participants had the desire to seek information; we did not ask about specific methods or sources of information. To improve the reliability of the measurement, future research should use a validated, standardized measurement instrument and examine the types of information-seeking behaviors they engaged in. Furthermore, because this study was intended to investigate whether adults with T2DM actively seek information on their own, we considered the quality of information to be a post-information-seeking issue. Therefore, this study did not take into account the risks of health information overload and exposure to misinformation. Information exposure in everyday life, such as through television, newspapers, and social media, may have a negative impact on patient behavior, and we would like to examine this in future research.

## Conclusion

Among Japanese adults with T2DM, there was an association between lower HbA1c values and a greater likelihood of engaging in information-seeking behavior. Feeling uneasy about the future at the time of diagnosis also seemed to lead to continuing feelings of anxiety when thinking about their T2DM in their ensuing lives. However, even if they had negative attitudes, some Japanese adults with T2DM were found to be able to engage in information-seeking behavior. Unlike previous studies, Japanese adults with T2DM having a sense of self-blame and who took a long time to seek medical help adopted information-seeking behavior.

## Data Availability

The datasets presented in this article are not readily available because requires approval from the Nara Medical University Ethics Committee. Requests to access the datasets should be directed to ino_rinri@naramed-u.ac.jp.

## Supplementary material

Supplementary Table S1 Questionnaire. ^*^Abbreviations of each question are indicated in parentheses. The abbreviations are used in other tables and a figure in this study.

Supplementary Table S2 Correlation coefficients between each question and HbA1c level. ^**^The correlation coefficient is significant (two-tailed) at the 1% level. ^*^The correlation coefficient is significant (two-tailed) at the 1% level.

Supplementary Table S3 Relationship between attributive variables and patient's attitudes. ^*^*p* < 0.05, n.a: no answer. Each questionnaire item (dependent variable) was analyzed with sex, age, household income, period until outpatient consultation, duration of diabetes, and HbA1c as independent variables. Dependent variables are listed above the rows listing *p* values and adjusted odds ratios, and are listed side-by-side for each group separated in the factor analysis.

Supplementary Table S4 Relationship between patient's information-seeking behavior and patient's attitudes. ^*^*p* < 0.05. The dependent variable in each case in this table is information-seeking behavior. The independent variables, sex, age, household income, period until outpatient consultation, duration of diabetes, and HbA1c, were analyzed at each step together with the specified additional independent variable as shown in each line of the table.

Supplementary Table S5 Relationship between patient's information-seeking behavior and patient's attitudes (Stratification at HbA1c 8%). ^*^*p* < 0.05, The dependent variable in each case in this table is information-seeking behavior. The independent variables, sex, age, household income, period until outpatient consultation, duration of diabetes, and HbA1c, were analyzed at each step together with the specified additional independent variable as shown in each line of the table.

Supplementary Table S6 Relationship between “Concerned about how I appear to others” and patient's other attitudes. ^*^*p* < 0.05, n.a: no answer. The dependent variable in each case in this table is “Concerned about how I appear to others.” The independent variables, sex, age, household income, period until outpatient consultation, duration of diabetes, and HbA1c, were analyzed at each step together with the specified additional independent variable as shown in each line of the table.

Supplementary Table S7 Relationship between patient's desire to discuss matters unrelated to DM and other attitudes. ^*^*p* < 0.05, n.a: no answer. The dependent variable in each case in this table is “Desire to discuss matters unrelated to DM.” The independent variables, sex, age, household income, period until outpatient consultation, duration of diabetes, and HbA1c, were analyzed at each step together with the specified additional independent variable as shown in each line of the table.

Supplementary Table S8 Examination of interactions between information-seeking behavior and attributive variables including an interactive item ^*^*p* < 0.05.

Supplementary Table S9 Examination of interactions between information-seeking behavior and attributive variables including an interactive item ^*^*p* < 0.05.

Supplementary Table S10 Relationship between patient's anxiety about diabetes and other attitudes. ^*^*p* < 0.05, n.a: no answer. The dependent variable in each case in this table is “Anxiety about diabetes.” The independent variables, sex, age, household income, period until outpatient consultation, duration of diabetes, and HbA1c, were analyzed at each step together with the specified additional independent variable as shown in each line of the table.

Supplementary Table S11 Relationship between patient's “undignified to ask others for help” and other attitudes. ^*^*p* < 0.05, n.a: no answer. The dependent variable in each case in this table is “Undignified to ask others for help.” The independent variables, sex, age, household income, period until outpatient consultation, duration of diabetes, and HbA1c, were analyzed at each step together with the specified additional independent variable as shown in each line of the table.

Supplementary Figure S1 Scatterplots of the relationship between patient's information-seeking behavior and HbA1c level.

Supplementary Figure S2 Scatterplots of the relationship between “Can focus on work or study” and HbA1c level.

Supplementary Figure S3 Scatterplots of the relationship between patient's self-blame and HbA1c levels.
