# Correction: Impact of negative attitudes and information-seeking behavior of adults with type 2 diabetes on treatment in Japan

**DOI:** 10.3389/fpubh.2025.1715200

**Published:** 2025-11-10

**Authors:** Takako Mohri, Sawako Okamoto, Tomoaki Imamura, Hitoshi Ishii

**Affiliations:** 1 Department of Diabetes and Endocrinology, Nara Medical University, Kashihara, Nara, Japan; 2 Department of Public Health, Health Management and Policy, Nara Medical University, Kashihara, Nara, Japan; 3 Department of Doctor-Patient Relationships, Nara Medical University, Kashihara, Nara, Japan

**Keywords:** diabetes mellitus, adherence, information-seeking behavior, self-management, HbA1c

Reference 7 was erroneously written as “American Diabetes Association Professional Practice Committee. Facilitating positive health behaviors and well-being to improve health outcomes: standards of care in diabetes-2025. *Diabetes Care*. (2025) 8(Supplement_1):S86–S127. doi: 10.2337/dc25-S005”. It should be “American Diabetes Association Professional Practice Committee. Facilitating positive health behaviors and well-being to improve health outcomes: standards of care in diabetes-2025. *Diabetes Care*. (2025) 48(Supplement_1):S86–127. doi: 10.2337/dc25-S005”.

Reference 11 was erroneously written as “日本糖尿病学会、糖尿病治療の目標と指、糖尿病診療ガイドライン 2024 東京、南江堂 p. 27-35 (Transl. by Mohri T) [The Japan Diabetes Society. “Goals and guidelines for diabetes treatment”. In: The Japan Diabetes Society, editor. *Japanese Clinical Practice Guideline for Diabetes 2024*. Tokyo: Nankodo Publishing Co., Ltd (2024). p. 27–35]”. It should be “日本糖尿病学会、糖尿病治療の目標と指針、糖尿病診療ガイドライン、2024、東京、南江堂、 p. 27–35 (Transl. by T. Mohri) [The Japan Diabetes Society. Goals and guidelines for diabetes treatment. In: The Japan Diabetes Society, editor. *Japanese Clinical Practice Guideline for Diabetes 2024*. Tokyo: Nankodo Publishing Co., Ltd (2024). p. 27–35]”.

Reference 28 was erroneously written as “Asendorpf JB. Social inhibition: a general-developmental perspective. In: Traue HC, Pennebaker JW, eds. *Emotion, Inhibition, and Health*. Seattle, WA: Hogrefe & Huber Publishers (1993). p. 80–99”. It should be “Denollet J. Interpersonal sensitivity, social inhibition, and type D personality: how and when are they associated with health? Comment on Marin and Miller (2013). *Psychol Bull*. (2013) 139:991–7. doi: 10.1037/a0033537”.

Reference 29 was erroneously written as “Denollet J. Interpersonal sensitivity, social inhibition, and type D personality: how and when are they associated with health? Comment on Marin and Miller (2013). *Psychol Bull*. (2013) 139:991–7. doi: 10.1037/a0033537”. It should be “Nefs G, Speight J, Pouwer F, Pop V, Bot M, Denollet J. Type D personality, suboptimal health behavior and emotional distress in adults with diabetes: results from diabetes MILES-The Netherlands. *Diabetes Res Clin Pract*. (2015) 108:94–105. doi: 10.1016/j.diabres.2015.01.015”.

Reference 32 was erroneously written as “石井 香織, 菅野 真由子, 木野 梢, 笹山 名月, 谷原 真一, 二次検査未受に影を与える要因の分析, 人間ドック (Ningen Dock), °2018, °33 巻, °3 号, °p. 471-477 (Transl. by Ishii K) [Ishii K, Kannno M, Kino K, Sasayama N, Tanihara S. Significant factors associated with failure to undergo secondary examination. *Ningen Dock*. (2018) 33:471–7]. doi: 10.11320/ningendock.33.471”. It should be “石井 香織, 菅野 真由子, 木野 梢, 笹山 名月, 谷原 真一, 二次検査未受診に影響を与える要因の分析, 人間ドック (Ningen Dock). (2018) 33:471–7 (Transl. by K. Ishii) [Ishii K, Kannno M, Kino K, Sasayama N, Tanihara S. Significant factors associated with failure to undergo secondary examination. *Ningen Dock*. (2018) 33:471–7]. doi: 10.11320/ningendock.33.471”.

Reference 45 was erroneously written as “石井 均, 山本 寿一, 久保 克彦, 他.糖尿病教育へのコンプライアンスに検する性格特性の影響 PF スタディによる検討糖尿病 (0021-437X)366 号 Page 461-468(1993.06) (Transl. by Ishii H) [Ishii H, Yamamoto T, Kubo K, Kubo E, Kato S, Yoshimasa Y, et al. Personality traits measured by picture-frustration study as positive and negative factors contributing to the short-term formation of good compliance in a diabetes education program. *J Japan Diab Soc*. (1993) 36:461–8]. doi: 10.11213/tonyobyo1958.36.461”. It should be “石井 均, 山本 壽一, 久保 克彦, 久保 永子, 加藤 純子, 吉政 康直, 他. 糖尿病教育へのコンプライアンスに対する性格特性の影響 PF スタディによる検討. 糖尿病 (0021-437X) 36 巻 6 号 Page 461-468 (1993.06) (Transl. by Ishii H) [Ishii H, Yamamoto T, Kubo K, Kubo E, Kato S, Yoshimasa Y, et al. Personality traits measured by picture-frustration study as positive and negative factors contributing to the short-term formation of good compliance in a diabetes education program. *J Japan Diab Soc*. (1993) 36:461–8]. doi: 10.11213/tonyobyo1958.36.461”.

Reference 52 was erroneously written as “松浦司．学歴学力が所得に与える影について．響について．経済論叢(京都大学)第 78第 3号, 111-129頁, 2006年 (Transl. by Okamoto S) [Matsuura T. The impact of social rank, educational status, academic performance on earnings. *Economic Ronso*. (2006) 78:111–29]. doi: 10.14989/125620”. It should be “松浦 司．階層・学歴・学力が所得にあたえる影響について. 経済論叢 (京都大学). 第 178 巻, 第 3 号 302–321頁, 2006 年. (Transl. by the journal 経済論叢) [Matsuura T. The effect of social class, schooling and ability on wage. Kronso (Kyoto University, Society of Economics's official academic journal). (2006) 178:302–21]. doi: 10.14989/125620”.

The original version of this article has been updated.

